# Circulating RNAs as Potential Biomarkers in Amyotrophic Lateral Sclerosis

**DOI:** 10.3390/ijms21051714

**Published:** 2020-03-03

**Authors:** Metka Ravnik-Glavač, Damjan Glavač

**Affiliations:** 1 Institute of Biochemistry, Faculty of Medicine, University of Ljubljana, Vrazov trg 2, 1000 Ljubljana, Slovenia; 2 Department of Molecular Genetics, Institute of Pathology, Faculty of Medicine, University of Ljubljana, Korytkova 2, 1000 Ljubljana, Slovenia

**Keywords:** amyotrophic lateral sclerosis, ALS, circulating biomarkers, circulating RNAs, blood, miRNA, lncRNA, circRNA, mRNA, ceRNET

## Abstract

Amyotrophic lateral sclerosis (ALS) is a complex multi-system neurodegenerative disorder with currently limited diagnostic and no therapeutic options. Despite the intense efforts no clinically applicable biomarkers for ALS are yet established. Most current research is thus focused, in particular, in identifying potential non-invasive circulating biomarkers for more rapid and accurate diagnosis and monitoring of the disease. In this review, we have focused on messenger RNA (mRNA), non-coding RNAs (lncRNAs), micro RNAs (miRNAs) and circular RNA (circRNAs) as potential biomarkers for ALS in peripheral blood serum, plasma and cells. The most promising miRNAs include miR-206, miR-133b, miR-27a, mi-338-3p, miR-183, miR-451, let-7 and miR-125b. To test clinical potential of this miRNA panel, a useful approach may be to perform such analysis on larger multi-center scale using similar experimental design. However, other types of RNAs (lncRNAs, circRNAs and mRNAs) that, together with miRNAs, represent RNA networks, have not been yet extensively studied in blood samples of patients with ALS. Additional research has to be done in order to find robust circulating biomarkers and therapeutic targets that will distinguish key RNA interactions in specific ALS-types to facilitate diagnosis, predict progression and design therapy.

## 1. Introduction 

Amyotrophic lateral sclerosis (ALS) belongs to a group of complex multi-factorial neurodegenerative diseases. It is characterized by the selective loss of upper and lower motor neurons [1]. This neuronal degeneration leads to progressive skeletal muscle atrophy and death by respiration failure after 2–5 years from the onset of symptoms. The disease, presenting in middle age, has an incidence of 2 per 100,000 persons per year. The familial ALS forms represent only around 5% of cases, while a majority of cases are sporadic forms, which are mostly phenotypically indistinguishable from familial forms suggesting the existence of common pathways at the basis of neuronal death [2]. Mutations in ALS-causing genes are associated with the disease in approximately 70% of familial forms of ALS (FALS) and in 15% of sporadic forms of ALS (SALS) [3]. Several pathological mechanisms have been demonstrated to induce neuronal death in ALS, from oxidative stress and mitochondrial impairment to glutamate excitotoxicity, growth factor deficiency, neuro-inflammation, defective axonal transport [4], RNA metabolism [5,6,7,8,9] and impaired brain energy metabolism [10]. Since the current diagnosis is based on clinical examination and electrophysiological measurements, the establishment of diagnosis is often delayed due to the clinical symptoms overlapping with alike neurological conditions at the early stage [11]. Often patients receive an alternative diagnosis before the diagnosis of ALS [12,13], and especially for the majority of patients with no genetic mutation or familial background. To date, there is no cure for ALS, so early detection of the disease in order to improve diagnosis, to predict and slow down disease progression and to develop treatment is an urgent need [14]. Identification of a panel of biomarkers that accurately reflect ALS pathology is a priority. It has been suggested that, besides genetics, certain environmental factors may contribute to the disease pathology through epigenetic changes [15,16]. Studies that showed disease discordance in monozygotic twin pairs support the idea that epigenetic mechanisms are involved in ALS disease development [17,18,19]. Among the main epigenetic mechanisms are methylation of DNA, differential expression of long non-coding RNAs (lncRNAs), micro RNAs (miRNAs), circular RNA (circRNAs), other non-coding RNAs and histone modifications. The effects of these epigenetic mechanisms are closely related, since for example DNA methylation can control the expression of miRNA, and vice versa—miRNA can regulate the expression of methylation enzymes. Furthermore, there is also a complex temporal and spatial cross-talk or endogenous competition between long non-coding RNA (lncRNA), circular RNA (circRNA), micro (miRNA), messenger RNA (mRNA) and other RNAs, which form RNA networks and shape the disease phenotypic outcomes. Recent research has placed an increasing importance on discovering RNAs networks, for both, understanding molecular mechanisms and developing novel diagnostic and prognostic markers as well as therapeutic targets.

**Figure 1:**
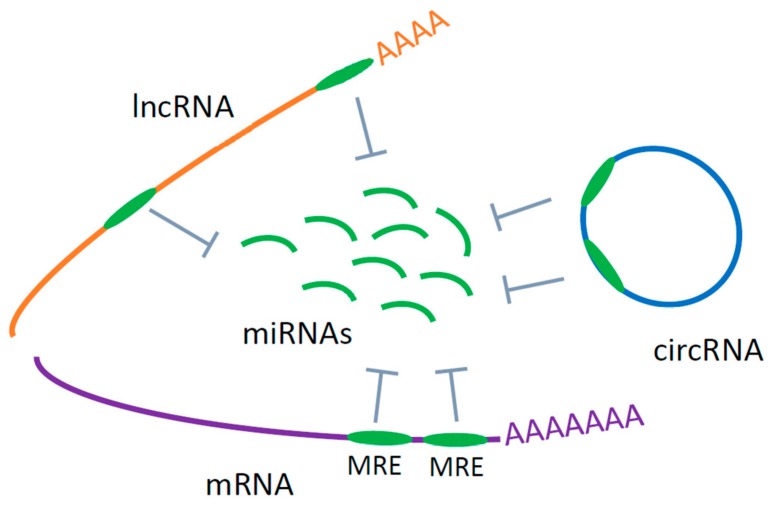
Competitive endogenous RNA (ceRNA) hypothesis: MicroRNAs (miRNAs) are commonly considered as active negative regulators of gene expression, decreasing the stability of target mRNAs or limiting their translation. miRNAs act through specific complementary binding of its seed region (usually 6–8 nt long) to the 3′ untranslated region (UTR) of target mRNA, called miRNA response elements (MRE). However, recent studies have shown that the interaction with mRNA is not unidirectional, but that also other endogenous RNAs, like lncRNAs, and circRNAs possessing the same MRE sequence (shown in the figure) can compete for the same pool of miRNA thereby regulating miRNA activity [23]. (green oval represents miRNA response elements (MRE)).

Despite the intense efforts, no clinically applicable biomarkers for ALS are yet established [12]. Development of biomarkers that are useful for early diagnosis and are selective for subgroups of disease as well as have prognostic potential and are indicators of treatment response are urgently needed. In addition, biomarkers should be minimally invasive and easily accessible from living patients. Most current research is thus focused, in particular, in identifying potential non-invasive RNA based circulating biomarkers for more rapid and accurate diagnosis and monitoring of the disease. Since ALS is a complex multi-factorial disease, no singular molecular species will be enough to enable the diagnosis or prognosis of ALS. Thus, an integrative approach based on several levels of RNA, including mRNA and non-coding RNAs, that form a potential biomarker signature is needed [20]. This approach has been already shown useful for other complex diseases, like cancers and neurodegenerative diseases [21,22]. In this review we have thus primarily concentrated on studies that have been foremost designed to find biomarkers for ALS based on mRNA, miRNA, lncRNA and circRNA in peripheral blood serum, plasma and cells, with little attempt to underlie the biology of the disease. We considered each RNA species separately as potential circulating biomarkers for ALS and in our conclusions we propose a potential integrated biomarker composed of the lncRNA/circRNA-miRNA-mRNA axis based on a competitive endogenous RNA network (ceRNET) hypothesis (see Figure 1 and Section 6) [22,23].

### Search Strategy and Criteria

We searched the PubMed and Google Scholar databases for articles in English. The main search terms included Amyotrophic Lateral Sclerosis, ALS, combined with RNA biomarkers and circulating RNA biomarkers. Since this is a relatively new research topic, no filter for the years was applied. We hand-searched the retrieved articles and selected the most relevant articles based on a subjective evaluation of their quality and relevance. Additional articles on specific topics were searched if needed.

## 2. mRNAs in ALS 

The dysregulated RNA mechanism is present in the familial and sporadic form of ALS. Abnormal GGGGCC hexanucleotide repeat expansion in the Chromosome 9 open reading frame 72 (C9orf72) is associated with 30–40% of familial cases and 1%–10% of sporadic cases of ALS [24]. This alteration causes the formation of R-loops and thus alters the RNA binding activity of translated C9orf72 protein [25,26]. Mutated superoxide dismutase (SOD1) was detected in 10%–20% of familial cases and in 2%–7% of sporadic ALS cases [27]. Alterations in SOD1 produce increased intracellular toxicity and trigger the apoptosis of the neuronal cells [28]. It was shown also that mutated SOD1 affects expression of two other mRNAs involved in neurodegeneration, vascular endothelial growth factor-A (VEGFA) mRNA and neurofilament (NEFL) mRNA [26]. Fused in sarcoma (FUS) RNA binding protein is a nuclear protein involved in splicing of RNAs and in the initiation of transcription [29]. Its prevalence in familial cases is about 5–7% and approximately 1% in sporadic cases [30,31]. Transactive response DNA binding protein (TDP-43) is involved in mRNA stability, RNA splicing, microRNA processing, and transport and local translation in neurons. Mutations in TDP-43 are present in about 3%–5% familial cases and in 1%–2% sporadic cases [31]. Both, FUS and TDP-43 are predominantly found in nucleus, but their mutations produce an export to the cytoplasm, formation of stress granules and neurodegeneration [32,33,34]. It was shown recently that different ALS-linked FUS mutations potentially caused disparate pathogenic pathways, but strikingly, nuclear import receptor Karyopherin-beta2 recovered the mutant defects [35]. Archbold et al. showed that several overlapping mechanisms regulate TDP-43 nuclear export [36]. It was revealed that the proteostasis system and the macroautophagy system are both severely involved in TDP-43 aggregation [37,38]. Studying TDP-43 structure, François-Moutal et al. reviewed how different domains and post-translational modifications may influence aggregation and deleterious behavior of TDP-43 [39].

Recently, Maniatis et al. performed comprehensive gene expression analysis using RNA sequencing in different regions of the spinal cord of a mouse ALS model over the course of disease and of a postmortem human ALS to detect transcriptomic changes that contribute to motor neuron loss. They found regional differences between microglia and astrocyte populations at early time points and confirmed altered expression of several known ALS genes e.g., *MATR3*, *KIF5A* and *PFN1* [40,41]. Furthermore, their data suggest that microglial dysfunction occurs well before onset of symptoms, precedes astroglial dysfunction in ALS and is proximal to motor neuron dysfunction. TREM2- and TYROBP-mediated signaling that modulates autophagy is an early step in the spatiotemporal ordering of disease-associated changes in microglial gene expression [41].

### mRNAs as Candidate Circulating Biomarkers of ALS 

Some comprehensive studies to detect differentially expressed mRNA in whole blood and peripheral blood mononuclear cells (PBMC) [42,43,44,45] and several studies on selected mRNAs potentially involved in ALS pathology [42,43,44,46,47,48,49] have been performed, however, no obvious candidate biomarkers have been yet identified. Vijayakumar et al. performed a systematic literature search and detected no reproducibility across different studies concerning the transcriptomic signature of ALS tissues [50]. The reasons for the lack of repeatability could be different study populations, different types of control subjects, different sample sources, different stages of the disease and the use of different methodological strategies [50]. The majority of detected differently expressed mRNAs have low or no ALS disease specificity. However, in the study of Kuzma-Kozakiewicz et al. using real-time qPCR and Western blotting PBMCs from 74 SALS patients with different clinical phenotypes, 65 blood donors (healthy control I) and 29 cases with other neurological diseases (disease control II) divided into subgroups IIA (atypical parkinsonism) and IIB (ALS-mimicking disorders) were investigated. KIF5C and KIFC3 expression were significantly lower and DCTN1 higher in SALS than in control I. KIF1B expression was significantly higher in SALS than in subgroup IIB, whereas DCTN1 and DCTN3 were higher in SALS than in subgroup IIA. Kinesin heavy chain isoform 5 (KIF5C) has critical roles in the developing brain, including organelle transport along microtubules [51]. Dynactin subunit 1 (DCTN1) is the largest subunit of the dynactin complex, an activator of the molecular motor protein complex dynein. Reduced levels of DCTN1 mRNA and protein have been detected in patients with sporadic amyotrophic lateral sclerosis, and mutations have been linked to disease [52]. KIF1B, Kinesin family member 1B is a motor protein that transports mitochondria and precursors of synaptic vesicle. Mutations in this gene cause Charcot–Marie–Tooth disease, type 2A1 [53]. Never the less, more studies are needed to find out whether the levels of KIF5C and DCTN1 may be useful in ALS diagnosis, and whether KIF1B expression may discriminate ALS from ALS-mimicking disorders [47]. Nachmany et al. detected a significant difference in mRNA expression levels of cytoplasmic FMR1 interacting protein 2 (CyFIP2) and RB binding protein 9 (RbBP9) genes in ALS compared to non-ALS peripheral blood leukocytes samples. CyFIP2 and RbBP9 proteins could be functionally related to cell cycle control and DNA damage mediated apoptosis [48]. CyFIP2 is expressed in the nervous system since it was found to directly interact with fragile X mental retardation protein (FMRP) and is present in mouse brain synaptosomal extracts [54]. We reported significant overexpression of apoptosis-associated tyrosine kinase, AAKT mRNA, a host gene of miR-338 and significant downregulation of the GTPase dynamin 2 (DNM2) gene in leukocytes of 84 SALS patients compared to healthy controls [55]. It has been shown in a neuroblastoma cell-line that the AATK gene produces neuronal differentiation [56]. In developing mouse brains, AATK was connected to cell death in mature neurons and to neurite extension in developing neurons [57]. DNM2 was also previously found mutated in Charcot–Marie–Tooth neuropathy type CMT2M, a motor and sensory neuropathy primarily affecting peripheral nerves [58] and in centronuclear myopathy (CNM), presenting with primary damage in skeletal muscles [59]. Loss of DNM2 in skeletal muscle initiates a chain of harmful parallel and serial events, involving dysregulation of lipid droplets and mitochondrial defects within altered muscle fibers, defective neuromuscular junctions and peripheral nerve degeneration [60]. Using deep sequencing of coding RNAs in monocytes of ALS patients compared to healthy controls a unique inflammation-related gene expression profile was detected, in which the most prominent mRNAs included IL1B, IL8, FOSB, CXCL1 and CXCL2 had higher expression levels in patients than in controls [61]. Gupta et al. reported that they found vascular endothelial growth factor-A (VEGF-A) and chemokine ligand (CCL2) genes upregulated in peripheral blood mononuclear cells in 50 Indian ALS patients compared with the same number of normal controls [46]. In a study that included 56 SALS patients and 20 healthy controls, Liguori et al. confirmed the importance of 12 genes whose mutations have been already identified as causative of SALS/FALS (*PFN1*, *TUBA4A*, *PARK7*, *SQSTM1*, *DCTN1*, *C9orf72*, *TMEM106B*, *ALS2*, *TRPM7*, *MATR3*, *SPG11* and *ATXN2*) [3,9,62,63,64,65,66] by detecting their differential expression (mRNAs) in the peripheral blood samples of SALS patients compared to controls [67]. They also identified differential expression of other potential candidate genes like galectin 3 (*LGALS3*), which is implicated in neuroinflammation and protein kinase C delta (*PRKCD*), which is activated in mitochondrial-induced apoptosis [67]. Additional studies are needed to find out if any of these or other ALS associated mRNAs will be possible to use as circulating biomarkers for ALS.

## 3. Micro RNAs 

MicroRNAs (miRNAs) are small 17–22 nucleotides (nt) long non-coding RNAs, which act as post-transcriptional regulators of gene expression either by causing the degradation of target mRNAs or the inhibition of their translation [68] through specific bounding of miRNA 6–8 nt long seed sequence to the 3′ untranslated region (UTR) of target mRNA [69]. Many miRNAs are evolutionarily conserved and have preferentially conserved interaction with most human mRNAs, which is indicative of their important biological functions [70]. Beside mRNA, miRNAs are also potent regulators of other miRNAs and non-coding RNAs, like lncRNAs and circRNAs. A single microRNA can regulate hundreds of RNA species, which makes miRNAs important regulators of cellular homeostasis [71]. MiRNAs are present in both intracellular and extracellular environments and in almost all biological fluids [72]. Extracellularly, miRNAs are detected within membrane vesicles and freely, forming complexes with other macromolecules. So far, more than 2500 miRNAs have been identified in the human genome (miRBase (www.mirbase.org)) [73] and for hundreds of them, the function in brain development and pathology have already been described [74].

### 3.1. MicroRNAs in Neurodegeneration and ALS

Several neurodegenerative diseases including ALS share numerous deregulated miRNAs, among them miR-9, miR-17, miR-21, miR-22, miR-25, miR-26, miR-29, miR-30, miR-34, miR-49, miR-98, miR-106, miR-107, miR-124, miR-125, miR-127, miR-128, miR-132, miR-132/212 cluster, miR-133b, miR-134, miR-136, miR-141, miR-146a, miR-153, miR-155, miR-181, miR-183, miR-183/96/182 cluster, miR-186, miR-193, miR-196, miR-200, miR-206, miR-210, miR-212,miR-221, miR-223, miR-320, miR-338, miR-365, miR-378, miR-494, miR-505, miR-512, miR-592, miR-let7 and miR-Let-7f-5p [75,76,77,78,79,80,81,82,83,84,85,86,87,88,89,90,91,92,93,94,95,96,97,98,99,100,101,102,103,104,105,106,107,108,109].

miRNAs have many characteristics to be promising biomarkers of human diseases. They have been shown to have high specificity, stability and recent studies have also indicated that miRNAs can be detected in biological fluids where they retain the expression profile of the original cells, including neurons [110].

### 3.2. miRNAs as Candidate Circulating Biomarkers of ALS

Numerous studies on miRNAs as potential circulating biomarkers for ALS have been published.

De Felice et al. detected several differentially expressed miRNAs in leukocytes from ALS patients including miR-149, miR-328, miR-338-3p, miR-451, miR-583, miR-638, miR-665 and miR-1275 [111]. 

CD14+CD16− monocytes isolated from ALS patients with SOD1 mutated familial form revealed a unique miRNA signature with miR-27a, miR-30b, miR-142-5p, miR-155, miR-223 and miR-532-3p highly overexpressed in an ALS patient comparing to healthy controls and patients with multiple sclerosis [109]. In addition, three of these miRNAs, miR-27b, miR-146a and miR-532-3p were upregulated also in sporadic ALS patients, and in monocytes and microglia from SOD1 mice [109].

In serum of sporadic ALS, Freischmidt et al. observed significant downregulation of TDP-43 binding miRNAs, miR-132-5p, miR-132-3p, miR-143-3p and miR-143-5p and let-7b [112].

Subsequently, miR-206 was reported elevated and as a potential biomarker in the serum of SALS patients [113].

Previously detected overexpression of miR-338-3p in blood leukocytes was confirmed in a larger cohort, and in addition, also in cerebrospinal fluid (CSF), serum and spinal cord obtained from SALS patients compared to healthy controls as well as to patients with other neurodegenerative disorders like Parkinson’s disease (PD), Alzheimer’s disease (AD) and Huntington disease (HD) [114].

Freischmidt et al. reported an miRNAs signature including miR-4745, miR-1915, miR-1825, miR-3613-3p, miR-3665, miR-3185, miR-4488, miR-3960, miR-4530, miR-1281, miR-4532, miR-4734, miR-477-5p, miR-4497, miR-3940-5p, miR-4466, miR-3196, miR-4270, miR-4507, miR-4505, miR-1469, miR-4741, miR-4787-5p, miR-371b-5p, miR-2861, miR-638, 149-3p, miR-4763-3p and miR-4516 that was significantly down-regulated in the serum of FALS and of presymptomatic mutation carriers compared to controls [115]. However, only two miRNAs, miR-1234-3p and miR-1825, were subsequently identified by the same group to be consistently downregulated in serum of sporadic ALS patients [116].

Takahashi et al. identified in the plasma of 48 SALS patients, compared to 47 healthy controls, miR-4649-5p up-regulated and miR-4299 down-regulated [117].

An miRNA panel of four under-expressed microRNAs, miR-183, miR-193b, miR-451 and miR-3935 was identified that distinguished among 83 SALS patients and 61 controls with high diagnostic accuracy of SALS (AUC 0.857). miR-183 also significantly discriminated between SALS patients and 24 Parkinson’s disease patients [118].

In plasma from 39 sporadic patients with spinal onset, miR-424 and miR-206 were found to be overexpressed compared to the same number of controls and their baseline expression correlated with clinical deterioration over time [119].

In 14 ALS patients (10 spinal, 4 bulbar) Tasca et al. measured the serum levels of muscle-specific miR-206, miR-1, miR-133a/b and miR-27a and found miR-206 and miR-133 significantly increased and miR-27a significantly reduced as compared to controls as well as between spinal vs. bulbar onset [120].

Thirty-seven brain-enriched miRNAs, inflammation-associated miRNAs, miRNAs highly enriched in muscle tissue and in cerebellum, and ubiquitous apoptosis-associated miRNAs were investigated in plasma of 250 patients with clinical diagnosis of either AD, frontotemporal dementia (FTD), PD or ALS. MicroRNA pairs and their combinations could differentiate all diseases from controls and from each other with high accuracy. Up-regulation of pairs miR-206/miR-338-3p, miR9*/miR-129-3p and miR-335-5p/miR-338-3p differentiated ALS from controls, while combinations miR-31/miR-206, miR-125b/miR-335-5p and miR-107/miR-491-5p differentiated ALS from AD, respectively [121].

miR-206 and miR-143-3p were increased and miR-374b-5p was decreased in the serum of SALS patients compared to controls and persisted during disease progression [122].

Serum miRNAs of 20 SALS and 3 FALS patients were compared to serum miRNAs of healthy controls, AD and multiple sclerosis patients. Seven microRNAs (miR-192-5p, miR-192-3p, miR-1, miR-133a-3p, miR-133b, miR-144-5p and miR-19a-3p) were identified as upregulated and six microRNAs (miR-320c, miR-320a, let-7d-3p, miR-425-5p, miR-320b and miR-139-5p) as downregulated in ALS patients in relation to healthy and ill controls [123].

We aimed to evaluate in leukocyte samples of 84 patients with sporadic ALS the differential expression of 10 miRNAs, for which some connection to ALS was shown previously in ALS culture cells, animal models or patients. We observed significant up-regulation across our patient cohort for miR-124a, miR-206, miR-9, let-7b, miR-638, miR-663a, miR-451, miR-132 and miR-338 [55].

Xu et al. isolated serum exosomes from ALS patients and healthy controls and compared the expression of miR-27a-3p. They found miR-27a-3p significantly down-regulated [124].

In serum from twenty sporadic ALS patients, a significant deregulation of miR-142-3p and miR-1249-3p was observed. miR-142-3p levels were found to negatively correlate with the ALS functional rating scale [125].

All the above described studies used as an miRNA detection approach combination of microarrays and qRT-PCR or qRT-PCR alone, however in three most recent studies, high-throughput next-generation RNA sequencing was applied [45,67,126].

In peripheral blood samples of SALS patients compared to healthy controls, results of analysis revealed that 38 miRNAs (let-7a-5p, let-7d-5p, let-7f-5p, let-7g-5p, let-7i-5p, miR-103a-3p, miR-106b-3p, miR-128-3p, miR-130a-3p, miR-130b-3p, miR-144-5p, miR-148a-3p, miR-148b-3p, miR-15a-5p, miR-15b-5p, miR-151a-5p, miR-151b, miR-16-5p, miR-182-5p, miR-183-5p, miR-186-5p, miR-22-3p, miR-221-3p, miR-223-3p, miR-23a-3p, miR-26a-5p, miR-26b-5p, miR-27b-3p, miR-28-3p, miR-30b-5p, miR-30c-5p, miR-342-3p, miR-425-5p, miR-451a, miR-532-5p, miR-550a-3p, miR-584-5p and miR-93-5p) were significantly downregulated in patients. It was also found that different miRNAs profiles characterized the bulbar/spinal onset and the progression rate of disease [67].

When compared between the ALS blood and healthy control blood, De Felice et al. identified 696 known and 49 novel miRNAs differentially expressed in ALS tissues. Most upregulated miRNAs were miR-224-3p, miR-5684, miR-4695-3p, miR-1296-5p, miR-224-5p, miR-153-3p, miR-10b-5p, miR-1, miR-3194-3p, miR-877-3p, miR-326 and miR-338-3p, and the most downregulated were miR-144-5p, miR-190a-5p, miR-218-5p, hsa-miR-125a-3p miR-143-3p, miR-144-3p, miR-618, miR-338-5p, miR-4423-3p, miR-542-5p, miR-199b-5p and miR-193a-5p [45].

Differentially expressed miRNAs were detected in extracellular vesicles (EVs) from the plasma of persons living with ALS (PALS) and healthy controls using high-throughput sequencing and droplet digital PCR (ddPCR). Results revealed elevated levels of 5 miRNAs and reduced levels of 22 miRNAs. Deregulated miRNAs most relevant to ALS included miR-9-5p, miR-183-5p, miR-338-3p and miR-1246. miR-15a-5p was identified for its diagnostic potential and miR-193a-5p for disability progression [126].

### 3.3. The Most Promising Potential Circulating miRNA Biomarkers and Their Physiological Roles

Although numerous potential miRNAs were proposed in several studies mentioned above as biomarkers in peripheral blood from ALS patients, there is not much overlap between each study. Most clinically relevant diagnostic and prognostic biomarkers, as well as treatment targets, would be those which are unable to detect disease at most early stages of its molecular pathogenesis. Vijayakumar et al. performed a systematic literature search and identified only a relatively few number candidates that were consistently identified as potential biomarkers across multiple independent studies. These candidate biomarkers are predominantly involved in dysfunction of skeletal muscle and of motor neurons and in inflammatory process [50].

Before the disease is clinically detectable and during its progression, the skeletal muscle of ALS patients undergoes futile cycles of re-innervation and denervation, along with motor neuron degeneration [127]. Muscle fibers are classified into two main metabolic types, slow-twitch and fast-twitch. Slow-twitch fibers are innervated by small-caliber axons and fast-twitch fibers are innervated by large-caliber axons. Studies revealed that the number of large-caliber axons is significantly lower in the spinal cord of ALS patients than in controls, whereas the number of small-caliber axons is maintained [128,129]. In the ALS mouse model, muscles enriched in slow-twitch fibers undergo denervation at later stages, compared with fast-twitch fibers [130] what suggests that, during the early stages of ALS pathogenesis, the muscle fibers and the motor neurons innervating them closely collaborate to counteract the skeletal muscle atrophy [131]. The role of microRNAs in the control of these crucial mechanisms has been investigated, though not yet conclusively clarified. A set of microRNAs enriched and specifically expressed in the skeletal muscle have been identified including miR-1, miR-133a, miR-133b, miR-206 and miR-27a [131]. Interestingly, each of these miRNAs has been more frequently detected also as deregulated circulating miRNAs in ALS patients compared to controls [55,109,113,119,120,122,123,124]. These miRNAs are involved in the regulation of different processes during skeletal muscle development. miR-1 is acting on transcription factor histone deacetylase (HDAC4) and paired box 7 (PAX7) and is required for muscle differentiation, while miR-133 promotes proliferation [132]. Williams and colleagues [133] originally investigated microRNA expression in the skeletal muscle during ALS progression in muscle tissues of symptomatic G93A-SOD1 transgenic mice. In this model, the upregulation of miR-206 coincided with the onset of neurological symptoms, since the transcriptional activation of miR-206 was activated in response to skeletal muscle denervation before clinical symptoms. miR-206 mediated its effects by suppressing muscular histone deacetylase 4 (HDAC4) protein levels. HDAC4 inhibition, in turn, induced the expression of fibroblast growth factor binding protein 1 (FGFBP1), which promoted re-innervation and regeneration within the neuromuscular junction [133]. It seems that miR-206 is not involved in the ALS pathogenesis, but is rather a potential early circulating biomarker of ALS progression and promising target for testing new treatments based on physiologic re-innervation self-healing responses.

miR-338-3p is another potential circulating miRNA for ALS, since it was found consistently upregulated in several studies [45,50,111,114,121,126]. Its role as a functional miRNA in controlling different molecular pathways has been reported. miR-338-3p is involved in neuronal proliferation, maturation and neurite outgrowth and in organization in the dentate gyrus, while also acting as a tumor suppressor in vivo [134,135,136,137]. miR-338-3p is also expressed in spinal cord oligodendrocytes, where it positively regulates oligodendrocyte differentiation from precursors into mature oligodendrocytes by repressing *Sox5* and *Hes6*, two maturation- and differentiation-inhibiting transcription factors [138,139]. Interestingly, miR-338-3p might also relate to the higher glutamate levels observed in ALS patients. Namely, one of putative targets of deregulated miR-338p is membrane-bound transporter protein SLC1A2/EAAT2, which clears the excitatory glutamate from the extracellular space at synapses in the CNS. Excitotoxicity thus caused by down-regulation of SLC1A2 /EAAT2 is thought to contribute to motor neuron death in ALS [140]. Li et al. recently showed that miR-338-3p binds glycogen phosphorylase (PYGB) and causes its diminished expression and regional accumulation of glycogen in the spinal cord of ALS mice. These findings provide new insights into the metabolic dysfunctions in the progression of ALS and into new potential therapeutic targets [141]. However, miR-338-3p deregulation was also observed in other neurodegenerative diseases including HD [142]. 

miR-183 was found dysregulated in peripheral blood samples of sporadic ALS patients in three different studies [67,118,126]. miR-183 family (including miR-183, miR-96 and miR-182) are highly conserved miRNAs with many revealed roles in different cancers, immunity and nervous system [143,144,145,146,147]. Jawaid et al. also showed that impaired biogenesis of miR-183/96/182 cluster influence age-related memory decline and that TDP-43 and FUS are involved in regulation of the biogenesis. In the postmortem brain of ALS patient’s miR-183/96/182 was decreased [148]. 

Maniatis et al. recently reported that microglial dysfunction occurs well before the onset of disease symptoms in an ALS mouse model [41]. Detection of circulating miRNAs biomarkers involved in neuroinflammation through microglial activation, dysregulation of immune-related genes and recruitment of monocytes to affected tissues represents important steps towards improving diagnosis and future treatment of ALS. Butovsky et al. already demonstrated that infiltration of peripheral inflammatory monocytes into the CNS played an important role in progression of ALS. Inflammation-related miRNAs including miRNA let-7a, let-7b, miR-27a, miR-146a, miR-451, miR-223, miR-142-5p, miR-532-3p and miR-155 were significantly upregulated in peripheral monocytes from SOD1 mice and ALS patients with familial and sporadic disease. In addition, MiR-27a was able to differentiate multiple sclerosis patients from patients with ALS [109]. Among miRNAs involved in neuroinflammation, miR-451 was revealed deregulated in blood samples of ALS patients in most studies [50,55,67,109,111,118]. 

miR-451 is involved in the regulation of various human physiological and pathological processes. It acts as a tumor suppressor gene in most cancer types. miR-451 can directly affect the biological functions of tumor cells but can also, upon secretion into the tumor microenvironment via exosomes, indirectly affect tumor cell invasion and metastasis [149]. It was shown that miR-451 is regulated in vivo by protein kinase AMP-activated catalytic subunit alpha 1 (AMPK) pathway and that AMPK/miR-451 loop has the ability to switch between proliferative and migratory pattern behavior of glioma cells [150,151]. It was also suggested that miR-451 might relieve chronic inflammatory pain by inhibiting microglia activation-mediated inflammation via targeting toll-like receptor 4 (TLR4) [152].

miRNAs let-7 were also found deregulated in ALS in several studies [55,67,109,112,123]. Let-7 family miRNAs are involved in multiple pathways contributing to cell survival and cell death and influence cancer risk and prognosis as well as neuroinflammation [153,154,155]. It was shown recently, that miR cluster MC-let-7a-1 ~ let-7d promotes glioma cell autophagy and apoptosis by repressing signal transducer and activator of transcription 3 (STAT3) [156].

Proinflammatory miR-125a/b has also been detected as circulating miRNA in ALS patients in more than one study [45,121]. Parisi et al. thoroughly investigated the role of miR-125b in the modulation of nuclear factor kappa B subunit (NF-κb) signaling in microglia. They identified a pathogenic mechanism in ALS microglia, in which miR-365 and miR-125b negatively regulated interleukin-6 (IL-6) and STAT3 pathway, respectively, causing an increase in tumor necrosis factor-alpha (TNFα) expression and switching microglia toward a detrimental phenotype [96,157].

In order to contribute to the diagnosis of ALS, it is important to detect and track miRNAs involved in early molecular manifestations of disease. No single miRNA of described deregulated miRNAs is specific for ALS. In addition, since ALS is a multi-systemic disease, a combination of several miRNAs will be needed to test simultaneously in order to reach higher specificity and accuracy of diagnosis. In some studies, combinations of several miRNAs have already shown a higher accuracy than single miRNAs in discriminating ALS from healthy controls or other neurological disorders [118,121,123]. Up-regulation of pairs miR-206/miR-338-3p, miR9*/miR-129-3p and miR-335-5p/miR-338-3p differentiated ALS from controls, while combinations miR-31/miR-206, miR-125b/miR-335-5p and miR-107/miR-491-5p differentiated ALS from AD, respectively [121]. 

miR-206, miR-133b, miR-27a, mi-338-3p, miR-183, miR-451, let-7 and miR-125b were most commonly deregulated in multiple studies reviewed above (Table 1). To test clinical potential of this miRNA panel, a useful approach may be to perform such analysis on larger multi-center scale using similar experimental design.

## 4. Long Non-Coding RNA

Long non-coding RNA (lncRNA) is another type of RNA molecule that could be investigated as circulating biomarkers in ALS. LncRNAs are transcripts greater than 200 bp in length with no or little translational potential. lncRNAs are classified according to the genomic position from which they are transcribed: intergenic RNAs (lincRNAs), intronic lncRNAs, antisense lncRNAs (aslncRNAs), bidirectional lncRNAs and enhancer RNAs (eRNAs). They can either silence or enhance the expression of a proximal gene at the level of epigenetics, transcription and post-transcription [158]. Lines of evidence revealed the role of dysregulated lncRNAs in cancer [159,160]. There is also increasing evidence of the importance of lncRNAs in development of brain, function, maintenance and differentiation of neurons, as well as in neurodegenerative diseases [161,162].

### 4.1. Long Non-Coding RNA in Neurodegeneration and ALS

In ALS, long non-coding transcripts have now also been found at the C9ORF72 locus. The C9ORF72 repeat expansion region can be transcribed bidirectional and both, sense and antisense C9ORF72 transcripts (C9ORF72-AS) are elevated in the brains of ALS patients where they form nuclear RNA foci. RNA foci were most abundant in the frontal cortex but also occurred in astrocytes, microglia and oligodendrocytes [163]. LncRNA, nuclear-enriched abundant transcript 1_2 (NEAT1_2T) contains a GC-rich sequence and is predominantly expressed in spinal motor neurons in an early phase of ALS [164]. A neurotoxic ataxin 2 antisense transcript ATXN2-AS with a CUG repeat expansion may also contribute to ALS pathogenesis [165].

### 4.2. LncRNAs as Candidate Circulating Biomarkers of ALS

To date, only one study reported differentially expressed lncRNAs in peripheral blood mononuclear cells (PBMCs) from ALS patients. Gagliardi et al. found in total of 293 dysregulated lncRNAs in sporadic ALS patients without detected mutation. The majority (184/293 transcripts) were antisense lncRNAs and mostly unknown. In patients with mutation in the *FUS* gene, 21 lncRNAs were identified, 11 of them were antisense. In TDP-43 mutated patients 7 antisense lncRNA was detected, and only one was already described, SNAP25-AS, antisense lncRNA of synaptic protein SNAP25. In ALS patients with SOD1 mutation, 2 novel antisense RNAs have been revealed, one of these is creatine kinase, mitochondrial 2 (CKMT2) antisense [44]. There is still a lot to be done in order to identify and understand the role of lncRNAs in ALS.

## 5. Circular RNAs 

Circular RNAs (circRNAs) represent yet another class of important regulatory non-coding RNAs [166,167,168]. They arise from back-splicing events during precursor mRNA processing. CircRNAs are resistant to RNA exonucleases and are thus highly stable in cells. CircRNAs may function as miRNA sponges and may sequester RNA-binding protein (RBP), and thus influence gene regulation. Each circRNA competitively binds multiple miRNAs and reduces their mRNA silencing potential [169]. When circRNAs bind to RBP they can act as scaffolds for protein complexes [170].

### 5.1. Circular RNAs in Neurodegeneration and ALS

Precise temporal and spatial regulation of gene networks tailors the development, homeostasis and stress response of the central nervous system (CNS). Moreover, it has been revealed that circRNAs are spatiotemporally regulated and dynamically expressed during brain development and can thus significantly influence development of CNS and diseases [168]. circRNAs have been already implicated in several neurodegenerative diseases, such as AD [171] and PD [172]. RNA-binding protein FUS, has been recognized as an important modulator of circRNA expression. Errichelli et al. observed an overall downregulation of circRNA expression in FUS_−/−_ mice and expression was dysregulated also in FUSR521C and FUSP525L human induced pluripotent stem cell-derived motor neurons. Since cognate linear transcripts showed no significant alteration in expression levels, circRNA deregulation can be attributed to altered splicing dynamics due to mutated or absent FUS. Whether this is the reason for altered circRNA expression also in human tissues, it remains to be determined [173]. These findings could have considerable implications for further research on circRNAs in ALS as mutations in FUS have been detected in ALS patients [3].

### 5.2. Circular RNA as Candidate Circulating Biomarkers of ALS

We investigated a microarray expression profile of circRNAs in leukocyte samples from SALS patients and age- and sex-matched healthy controls and identified 425 differentially expressed circRNAs. We selected 10 of them based on the function of the hosting gene for qPCR validation. The expression of 7/10 circRNAs was significant in a larger cohort of ALS patients. Four of them showed the highest significance as well as clinical relevance [174]. hsa_circ_0000567 is located in SETD3 gene, the product of which is histone methyltransferase that regulates muscle differentiation in mouse [175]. hsa_circ_0023919 is located in the *PICALM* gene that is involved in clathrin-mediated endocytosis at neuromuscular junctions [176]. hsa_circ_0023919 sequence contains two binding sites for hsa-miR-9 [177]. The upregulation of miR-9 was confirmed in both mouse model of ALS [178] and in human blood samples of ALS patients [55]. *TNRC6B*, a host gene of hsa_circ_0063411, guides Ago mediated gene silencing [179]. It contains one binding site for miR-647 [177]. Some connection between miR-647 and ALS was already detected in spinal cord samples from ALS patients where miR-647 was absent compared to controls [180]. hsa_circ_0088036 is located in sushi domain containing 1 (SUSD1) gene that is potentially associated with ALS [181]. In addition, hsa_circ_0023919, hsa_circ_0063411 and hsa_circ_0088036 also had AUC > 0.95, and sensitivity and specificity for the optimal threshold point >90%, showing their potential for using them as diagnostic biomarkers [174].

## 6. Conclusions and Future Perspectives

**Figure 2:**
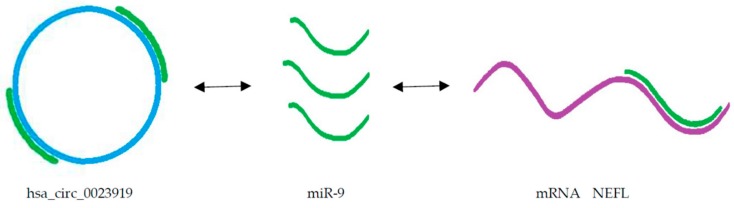
Potential circRNA–miRNA–mRNA axis for amyotrophic lateral sclerosis (ALS). This axis represents endogenous competition between circRNA hsa_circ_0023919 and mRNA *NEFL* for binding of miR-9. circRNA hsa_circ_0023919 sponges miR-9, which regulates metabolism of neurofilament (NEFL) [174,183].

ALS is a complex multi-system neurodegenerative disorder with currently limited diagnostic and no therapeutic options. Early diagnosis of ALS is, therefore, most important for rapid identification and management of the disease. Most studies to date have investigated miRNAs as potential circulating biomarkers for ALS. From these studies, a panel of the most promising potential circulating miRNAs with physiological roles in ALS could be deduced (Table 1). However, other types of RNAs (lncRNAs, circRNAs and mRNAs) that together with miRNAs represent RNA networks, have not been yet extensively studied in blood samples of patients with ALS. The RNA network represents a large-scale regulatory network across the transcriptome that plays important roles in maintaining the homeostasis, while the disruption of this network leads to pathological conditions. Molecular biomarkers based on specific RNA networks would have more potential to successfully distinguish between different neurological conditions. Specific RNA networks based on cross-talk between lncRNA/circRNA, miRNA and mRNA have recently attracted more attention [182]. Salmena et al. proposed the hypothesis about competing endogenous RNAs (ceRNAs) (Figure 1). According to this hypothesis, different RNA molecules, including lncRNAs, circRNAs and mRNA act as miRNA sponges to inhibit miRNAs from binding to their target sites. RNA transcripts that share the same miRNA response element (MRE) can competitively inhibit the function of miRNA and then influence the expression of relevant mRNAs [23]. Based on the ceRNA hypothesis, a cross-talk is going on between RNA transcripts with identical MREs regulating their reciprocal expression levels. Because these interactions are interconnected an aberrant expression of any component of network could interrupt the complex regulatory circuitry, resulting in the development of disease. Since all these RNAs can be detected in blood, the lncRNA/circRNA–miRNA-mRNA axes may represent innovative circulating biomarkers. Such ceRNA axes and networks (ceRNETs) based on computational platforms and experimental approaches have already been proposed for several cancers and neurodegenerative diseases [21,22], but not yet for ALS. Extensive investigation of the role of circulating mRNAs, lncRNAs and circRNAs is therefore needed to detect RNA networks specific for disease type and progression. We have recently reported high diagnostic potential of circRNA hsa_circ_0023919 in blood samples of SALS patients [174]. hsa_circ_0023919 contains two binding sites for miR-9 [177]. Hawley et al. have shown that human neurofilament (NEFL) 3’UTR had two sites for miR-9 binding and that miR-9 was capable of reducing the expression of *NEFL*, and intermediate neurofilaments were observed in motor neurons of SALS patients [183]. Deduced from these results one potential example of biomarker based on circRNA-miRNA-mRNA axis for ALS could be: hsa_circ_0023919—miR-9—m*NEFL*, where circRNA hsa_circ_0023919 sponges miR-9, while miR-9 regulates metabolism of intermediate filaments (NEFL) observed in ALS motor neurons [174,183] (Figure 2). A lot still must be done in order to find robust circulating biomarkers and therapeutic targets that will distinguish key ceRNA interactions in specific ALS-types to facilitate diagnosis, predict progression and design therapy.

## Figures and Tables

**Table 1:** The most promising potential circulating miRNA biomarkers.

miRNA	Sample	Level of miRNA	Reference
miR-206	Leukocytes, plasma, serum	increased	[55,113,119,120,122]
miR-133b	serum	increased	[120,123]
miR-27a	CD14+CD16- monocytes, serum exosomes	increased, decreased	[109,120,124]
mi-338-3p	leukocytes, plasma, plasma extracellular vesicles	increased	[45,55,111,114,121,126]
miR-183	leukocytes, plasma extracellular vesicles	decreased	[67,118,126]
miR-451	leukocytes, peripheral monocytes	decreased, increased	[55,67,109,111,118]
let-7	serum, leukocytes, peripheral monocytes	decreased, increased	[55,67,109,112,123]
miR-125	leukocytes, plasma	decreased	[45,121]
